# Experimental and Theoretical Investigation of the Structural and Opto‐electronic Properties of Fe‐Doped Lead‐Free Cs_2_AgBiCl_6_ Double Perovskite

**DOI:** 10.1002/chem.202004902

**Published:** 2021-03-22

**Authors:** Sachin Thawarkar, Sachin R. Rondiya, Nelson Y. Dzade, Nageshwar Khupse, Sandesh Jadkar

**Affiliations:** ^1^ Department of Physics Savitribai Phule Pune University Pune 411007 India; ^2^ School of Chemistry Cardiff University Cardiff CF10 3AT Wales United Kingdom; ^3^ Centre for Materials for Electronic Technology Dr. Homi Bhabha Road Pune 411008 India

**Keywords:** band gaps, density functional calculations, iron, perovskites, photoconductivity

## Abstract

Lead‐free double perovskites have emerged as stable and non‐toxic alternatives to Pb‐halide perovskites. Herein, the synthesis of Fe‐doped Cs_2_AgBiCl_6_ lead‐free double perovskites are reported that display blue emission using an antisolvent method. The crystal structure, morphology, optical properties, band structure, and stability of the Fe‐doped double perovskites were investigated systematically. Formation of the Fe‐doped Cs_2_AgBiCl_6_ double perovskite is confirmed by X‐ray diffraction (XRD) and X‐ray photoelectron spectroscopy (XPS) analysis. XRD and thermo‐gravimetric analysis (TGA) shows that the Cs_2_AgBiCl_6_ double perovskite has high structural and thermal stability, respectively. Field emission scanning electron microscopy (FE‐SEM) analysis revealed the formation of dipyramidal shape Cs_2_AgBiCl_6_ crystals. Furthermore, energy‐dispersive X‐ray spectroscopy (EDS) mapping shows the overlapping of Cs, Bi, Ag, Fe, and Cl elements and homogenous incorporation of Fe in Cs_2_AgBiCl_6_ double perovskite. The Fe‐doped Cs_2_AgBiCl_6_ double perovskite shows a strong absorption at 380 nm. It extends up to 700 nm, suggesting that sub‐band gap states transition may originate from the surface defect of the doped perovskite material. The radiative kinetics of the crystals was studied using the time‐correlated single‐photon counting (TCSPC) technique. Lattice parameters and band gap value of the Fe‐doped Cs_2_AgBiCl_6_ double perovskites predicted by the density functional theory (DFT) calculations are confirmed by XRD and UV/Visible spectroscopy analysis. Time‐dependent photo‐response characteristics of the Fe‐doped Cs_2_AgBiCl_6_ double perovskite show fast response and recovery time of charge carriers. We believe that the successful incorporation of Fe in lead‐free, environmentally friendly Cs_2_AgBiCl_6_ double perovskite can open a new class of doped double perovskites with significant potential optoelectronics devices fabrication and photocatalytic applications.

## Graphical abstract

**Doubling up**: The synthesis and first‐principles density functional theory (DFT) calculations of Fe‐doped, lead‐free dipyramidal shape Cs_2_AgBiCl_6_ crystals for photodetector applications are presented. The results of systematic investigation of the crystal structure, morphology, optical properties, band structure, and stability of these materials are discussed.

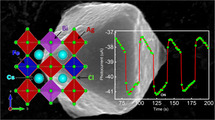

## Introduction

Lead halide A^I^Pb^II^X_3_ (A=CH_3_NH_3_, Cs; X=Cl, Br, I) perovskites have been established as efficient materials for photovoltaic (PV) and opto‐electronic applications with certified power conversion efficiencies (PCEs) exceeding 23 %.[^1^, ^2^, ^3^, ^4^, ^5^, ^6^] This has been attributed mainly to their outstanding intrinsic high absorption coefficient, suitable band gap, and high charge carrier mobility.[^7^, ^8^, ^9^, ^10^] Despite these wonderful properties and demonstrated high PCEs, the large scale deployment of these materials is limited by the lead contents toxicity and the intrinsic thermal and moisture instability.^[11]^ Significant efforts have been made to reduce the toxicity issues and improve the stability of lead halide perovskites. Various strategies have been designed and developed for the replacement of Pb. One such approach is replacing the Pb with the substitution of group IV metals with less toxicity, such as Ge^2+^ and Sn^2+^.[^12^, ^13^, ^14^, ^15^, ^16^] Unfortunately, these elements’ chemical stability is poor because they undergo facile oxidation from 2+ states to 4+ oxidation states.[^17^, ^18^] Another promising approach is hetero‐valent metal substitution. In this approach, two divalent Pb^2+^ ions are replaced with a pair of one trivalent B^3+^ and monovalent B^+^ cation, leading to the formation of A_2_B^I^B^III^X_6_ double perovskite materials, which persist the 3D crystal structure and the charge neutrality of perovskite.[^19^, ^20^, ^21^, ^22^, ^23^] Furthermore, incorporating another new element into the double perovskite structure guides the optical and structural properties depending on chemical compositions.^[24]^ Inorganic double perovskites A_2_B^I^B^III^X_6_ having variants A=Cs^+^, Rb^+^; B^I^=Ag^+^, Na^+^, K^+^, Li^+^; B^III^=Bi^3+^, Sb^3+^, In^3+^; X=Cl^−^, Br^−^, I^−^ show an alternating arrangement of B^I^ and B^III^ ions on the octahedral sites (rock salt).[^25^, ^26^, ^27^, ^28^, ^29^] Therefore, double perovskites materials are more attractive as they do not contain toxic Pb^2+^ ions and have good thermal and chemical stability and band gap tunability. For example, bismuth halide double perovskites such as Cs_2_AgBiCl_6_ and Cs_2_AgBiBr_6_ have indirect band gaps of 2.77 eV and 1.95 eV, respectively.[^30^, ^31^, ^32^, ^33^, ^34^] Bandgap tunability has also been achieved by doping Mn^2+^, Sb^3+^, and In^3+^ into the Cs_2_BiAgBr_6_ lattice.[^35^, ^36^, ^37^, ^38^] For example, Locardi et al.^[25]^ have reported high photoluminescence quantum yield (PLQY) and improved the visible light emission properties for Mn‐doped Cs_2_AgInCl_6._ This demonstrates that doping is an effective technique to achieve superior opto‐electronic properties and reduced defect state density in double perovskites. Both are essential to fabricate highly efficient opto‐electronic devices. There are, however, limited studies dedicated to the comprehensive characterization of the absorption, emission, recombination dynamic processes in double perovskites. In the present study, we report the synthesis of a high‐quality, lead‐free, and low‐cost Fe‐doped Cs_2_AgBiCl_6_ double perovskite via antisolvent method. The absorption, emission, and recombination dynamic properties were compressively characterized using time‐resolved photoluminescence spectroscopy (TR‐PL). Photoconductivity measurements under visible light with a standard photoelectrochemical (PEC) cell arrangement, demonstrates that the Fe‐Cs_2_AgBiCl_6_ samples exhibits high photocurrent. The prepared double perovskite materials not only retain their optical properties, but also show good thermal and electronic stability, which are helpful to make devices. This work provides novel insights into the structure‐property relationships in lead‐free double perovskites and offers new strategies for the development of advanced perovskite devices.

## Experimental Section

### Materials and chemicals

Cesium(I) chloride (CsCl, Sigma Aldrich, 99 %), silver(I) chloride (AgCl, Sigma Aldrich, 99.99 %), bismuth(III) chloride (BiCl_3,_ Sigma Aldrich 99 %), Ferric(III) chloride (FeCl_3_, Sigma Aldrich, 99.9 %), dimethyl sulfoxide (DMSO, Sigma Aldrich, 99.9 %) and isopropanol (Sigma Aldrich 99 %). All the precursors were used as received without further purifications.

### Synthesis of Cs_2_AgBiCl_6_ and Fe‐doped Cs_2_AgBiCl_6_ double perovskite

Undoped and Fe‐doped Cs_2_AgBiCl_6_ double perovskites were synthesized using the antisolvent method. The schematic representation of the synthesis protocol used for the synthesis is shown in Scheme S2 (Supporting Information). For the undoped sample, salts of 0.2 mmol (101.1 mg) CsCl, 0.1 mmol (42.9 mg) AgCl and 0.1 mmol (94.5 mg) BiCl_3_ were dissolved in 5 mL DMSO to form a precursor solution. After that, under vigorous stirring, 100 μL of the precursor solution was injected into 5 mL isopropanol. The mixed solution was then centrifuged at 3000 rpm for 3–6 min to remove large crystals. For the doped samples, FeCl_3_ was used as the Fe precursor. For 3 % and 6 % doping of Fe into Cs_2_AgBiCl_6_ double perovskites, 0.006 mm (2.84 mg) and 0.012 mm (5.68 mg) FeCl_3_ was taken along with the rest of the precursors, respectively. The protocol for the synthesis of doping was similar as described above.

### Characterization of Cs_2_AgBiCl_6_ and Fe‐doped Cs_2_AgBiCl_6_ double perovskite

UV/Visible spectra of the undoped and Fe doped Cs_2_AgBiCl_6_ double perovskites were recorded using UV‐1800 spectrophotometer (SHIMADZU, Japan) in isopropanol. The surface morphology and shape of the Fe doped Cs_2_AgBiCl_6_ double perovskite material were investigated under field emission scanning electron microscopy (FE‐SEM) using FEI Nova NanoSEM 450, EDS: Bruker X Flash 6130 instrument. Samples were made via drop‐casting on the silicon wafer and dried under reduced pressure for 12 h. Transmission electron microscopy (TEM) images were obtained from high‐resolution transmission electron microscopy (HR‐TEM) TALOS F‐200x; a drop of the sample was placed on a carbon‐coated copper grid dried in the dark for the night. The powdered X‐ray diffraction (XRD) patterns were measured on XRD, Bruker AXS D8 Advance using Cu_Kα_ radiation within a range of 20–80°. Thermogravimetric analysis (TGA) was recorded using a TGA‐50 Shimadzu under nitrogen atmosphere. The experiment was performed from 30 to 1000 °C with a heating rate of 10 °C min^−1^. Time‐resolved photoluminescence (TRPL) spectra were measured using a time‐correlated single‐photon counting (TCSPC) system by Edinburgh EPLED‐330. X‐ray photoelectron spectroscopy (XPS) studies were carried out using Thermo Scientific, K‐Alpha^+^, UK machine with a resolution of 0.1 eV. The machine can achieve vacuum >10^−9^ torr, and we have recorded XPS spectra for the specific element using Al_Kα_ (1486.6 eV) radiation. The XPS signals were obtained after several scans deployed in the acquisition process. The binding energy was corrected for specimen charging through referencing C 1s to 284.6 eV. The photo‐response of the Fe doped Cs_2_AgBiCl_6_ double perovskite was performed using a conventional 3‐electrode system.

### Density functional theory (DFT) details

The electronic structure calculations were performed using density functional theory (DFT) within periodic boundary conditions as implemented in the Vienna Ab initio Simulation Package (VASP).[^39^, ^40^, ^41^] The Perdew–Burke–Ernzerh of (PBE) functional^[42]^ was used for geometry optimizations and stability, while for electronic structures and optical calculations, the screened hybrid functional HSE06 with 25 % Hartree–Fock exchange was used, with the addition of spin‐orbit effects (HSE06+SOC).^[43]^ The valence and core electrons interactions were described with the projected augmented wave (PAW) method.^[44]^ A 3×3×3 Γ‐centered Monkhorst–Pack^[45]^ k‐mesh and a 600 eV plane‐wave cut off were used for structure optimization, while a tighter k‐mesh of 5×5×5 was used for the electronic structure calculations.

### Photoelectrochemical (PEC) cell assembly

Scheme S1 in the Supporting Information shows the schematic of the PEC cell employed in the present study. Three electrodes were placed inside the cell; synthesized Fe‐doped Cs_2_AgBiCl_6_ double perovskite film act as working electrode (WE), whereas platinum foil and saturated calomel electrode (SCE) were used as a counter electrode (CE) and a reference electrode (RE), respectively. 0.2 m Na_2_SO_4_ was used as the electrolyte. Potentiostat (Metrohm Autolab: PGSTAT302N) and 150 W Xenon Lamp (PEC‐L01) with illumination intensity of 100 mW cm^−2^ (AM 1.5) were used to record the *I–V* characteristics.

## Results and Discussion

### X‐ray diffraction (XRD) analysis

**Figure 1:**
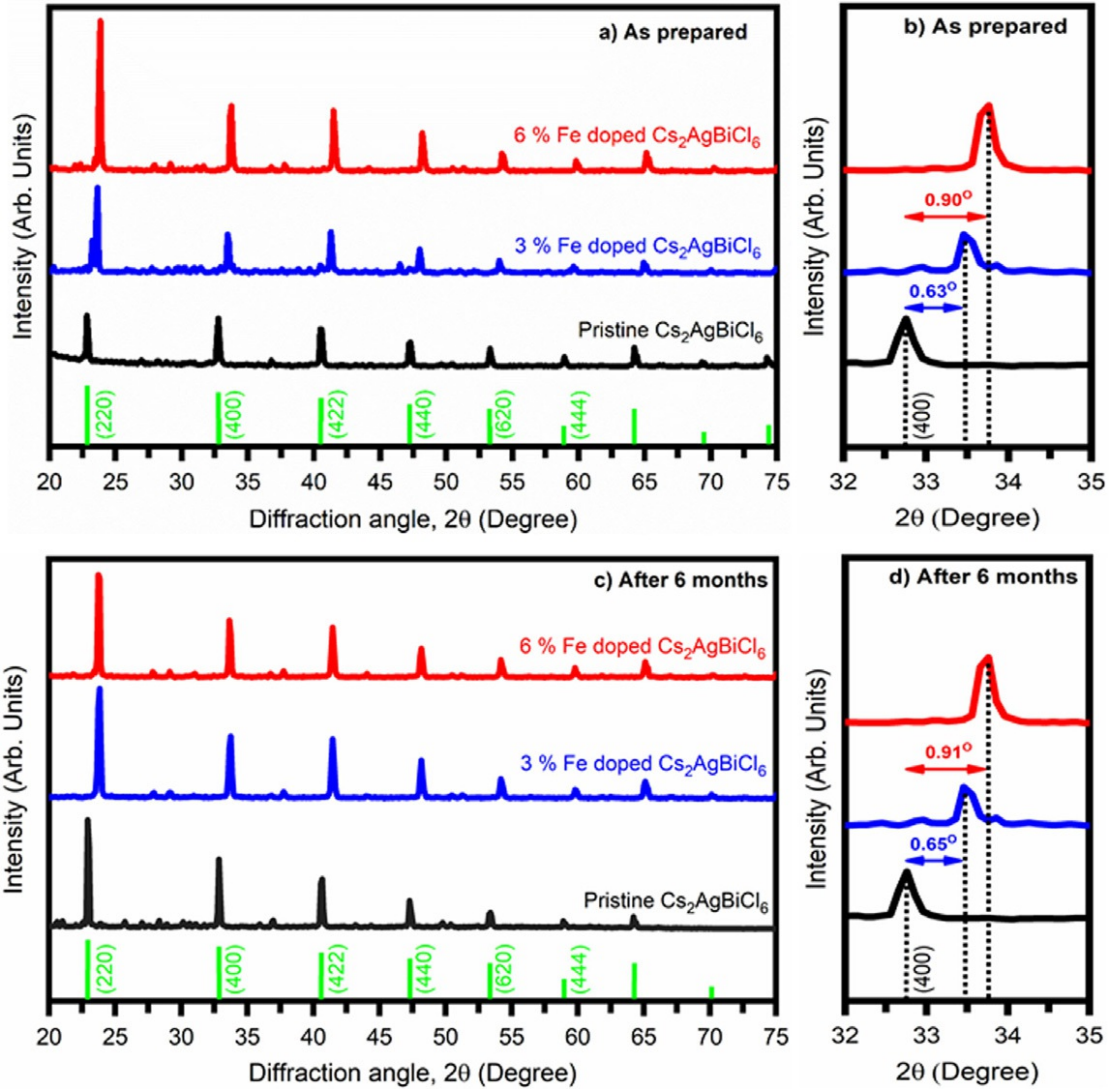
(a) XRD pattern for undoped and Fe‐doped Cs_2_AgBiCl_6_ double perovskites with different Fe concentrations, (b) Shifting of 2*θ* degree of (400) plane, (c) XRD pattern of undoped and Fe‐doped Cs_2_AgBiCl_6_ double perovskites with different Fe concentrations taken after six months exposure under normal temperature and pressure and (d) Zoomed‐in XRD patterns for clear visualization of (400) diffraction peak.

To perform the synthesis of Fe‐doped Cs_2_AgBiCl_6_ double perovskite at room temperature, we implemented a modified antisolvent method, which avoids the conventional heating, injection methods, and inert gas conditions. Scheme S2 (Supporting Information) shows the actual photographs of the Fe‐doped Cs_2_AgBiCl_6_ double perovskite under ordinary light and blue emission at 365 nm UV light excitation. A Cs_2_AgBiCl_6_ double perovskite is composed of Cs^+^ ion at the center of cuboctahedra with alternating [BiCl_6_]^−3^ and [AgCl_6_]^−5^ octahedral unit, which leads to the formation of a 3D network.^[31]^ Figure 1 a shows the XRD pattern of the Fe‐doped Cs_2_AgBiCl_6_ double perovskite. The presence of multiple diffraction peaks indicates that the Fe‐doped double perovskites are polycrystalline. Major diffraction peaks are observed in the XRD pattern at 2*θ*=23.63°, 33.36°, 41.27°, 47.98°, 54.00°, and 59.60°, which correspond to (220), (400), (422), (440), (620) and (444) diffraction planes of standard cubic double perovskite structure with a Fm$\bar{3}$ m space group and lattice parameter a 10.91 Å. These results are consistent with previous literature reports,[^20^, ^21^, ^27^] confirming the formation of Fe doped and undoped Cs_2_AgBiCl_6_ double perovskite via the antisolvent method. A careful analysis of the XRD pattern of the Fe‐doped Cs_2_AgBiCl_6_ double perovskite reveals a shift of all diffraction peaks towards a higher diffraction angle and a subsequent increase in the diffraction intensity with increasing Fe doping concentration. For example, the (400) diffraction peak is shifted by 0.63° and 0.90° relative to the undoped Cs_2_AgBiCl_6_ double perovskite for 3 % and 6 % Fe‐doping, respectively (see Figure 1 b). The shifts in diffraction peak and the increasing intensity indicate that the incorporation of smaller Fe^3+^ ion (0.63 Å) in place of larger Bi^3+^ ion (1.03 Å) induces contractions in the lattice. The lattice parameter contracted from 10.91 Å for the undoped to 10.67 Å and 10.60 Å for the 3 % and 6 % Fe‐doped Cs_2_AgBiCl_6._ We have observed an XRD peak at 46.42° in the 3 % Fe‐doped Cs_2_AgBiCl_6_ samples, which may be due to AgCl. The diffraction intensity of all the Fe‐doped samples is significantly high. The full width at half‐maximum is narrower, suggesting that the Fe‐doped double perovskites have excellent crystallinity. The interplanar *d*‐spacings (d) calculated from the diffraction peak positions (2*θ*) of the different diffraction planes (hkl) for the Fe‐doped Cs_2_AgBiCl_6_ double perovskite are displayed in Table S1 in the Supporting Information. It shows the clear differences between the structural properties (crystallite size, strain, interplanar distance, and lattice parameters) of the pristine, 3 %, and 6 % doping of Fe into Cs_2_AgBiCl_6_ double perovskites (Table 1). The contraction in the d‐values for the 3 % and 6 % Fe‐doped Cs_2_AgBiCl_6_ double perovskites relative to the undoped counterpart indicates the successful doping of Fe into Cs_2_AgBiCl_6_ double perovskite. We think that with Fe^3+^ doping, the [BiCl_6_]^3−^ octahedra may be replaced by [FeCl_6_]^3−^ octahedra in the Cs_2_AgBiCl_6_ double perovskite lattice. There are three possibilities by which Fe^3+^ ion can incorporate into a Cs_2_AgBiCl_6_ crystal lattice. In Cs_2_AgBiCl_6_ double perovskite, [BiCl_6_]^3−^ octahedra, [AgCl_6_]^5−^ octahedra and Cs^+^ ion of A‐site can be replaced by [FeCl_6_]^3−^ octahedra leading to the formation Fe‐doped Cs_2_AgBiCl_6_ lattice. However, due to the large electronegativity difference between Fe (1.83) and Cs (0.79) as well as the charge difference between Fe (3+) and Cs (+), the possibility of replacement of Cs by Fe ion in Cs_2_AgBiCl_6_ lattice is ruled out. On the other hand, the electronegativity of Fe is close to that of Ag (1.93) and Bi (2.0) and the charge balance is significantly maintained by Bi^3+^ and Fe^3+^ compared to that of Bi^3+^ and Ag^+^, therefore [FeCl_6_]^3−^ octahedra is expected to replace the [BiCl_6_]^3−^ octahedra is more dominant in the formation of Fe‐doped Cs_2_AgBiCl_6_ lattice.

**Table 1:** Crystallite size, strain, interplanar distance, and lattice parameters of pristine, 3 %, and 6 % doping of Fe into Cs_2_AgBiCl_6_ double perovskites.

Sample	Crystallite size [nm]	Strain	d_(hkl)_ [Å]	a=b=*c* [Å]
Pristine Cs_2_AgBiCl_6_	D_1_=48.76 D_2_=37.76	S_1_=3.59×10^−3^ S_2_=3.25×10^−3^	d_1_=3.89 d_2_=2.73	a_1_=b_1_=c_1_=11.00 a_2_=b_2_=c_2_=10.91
3 % of Fe doped Cs_2_AgBiCl_6_	D_1_=48.83 D_2_=43.68	S_1_=3.46×10^−3^ S_2_=3.46×10^−3^	d_1_=3.76 d_2_=2.68	a_1_=b_1_=c_1_=10.63 a_2_=b_2_=c_2_=10.67
6 % of Fe doped Cs_2_AgBiCl_6_	D_1_=45.11 D_2_=39.57	S_1_=3.43×10^−3^ S_2_=3.02×10^−3^	d_1_=3.73 d_2_=2.65	a_1_=b_1_=c_1_=10.51 a_2_=b_2_=c_2_=10.60

To investigate the structural stability of the Fe‐doped Cs_2_AgBiCl_6_ double perovskites, XRD measurements were carried out after six months on the same samples after exposing them to ambient environmental conditions without any encapsulation. Figure 1 c shows the XRD pattern of the undoped and Fe‐doped Cs_2_AgBiCl_6_ samples taken after six months of exposure under normal temperature and pressure. Figure 1 d shows the zoomed‐in XRD patterns for clear visualization of the (400) diffraction peak. It has been observed that there is no significant change in diffraction intensity of all diffraction peaks, as well as no significant change or shift in the diffraction angles after six months of ambient exposure of the Fe‐doped Cs_2_AgBiCl_6_ perovskite. These results suggest that the Fe‐doped Cs_2_AgBiCl_6_ nanocrystals have excellent stability after six months of exposure to ambient environmental conditions.

### Thermo‐gravimetric analysis (TGA)

**Figure 2:**
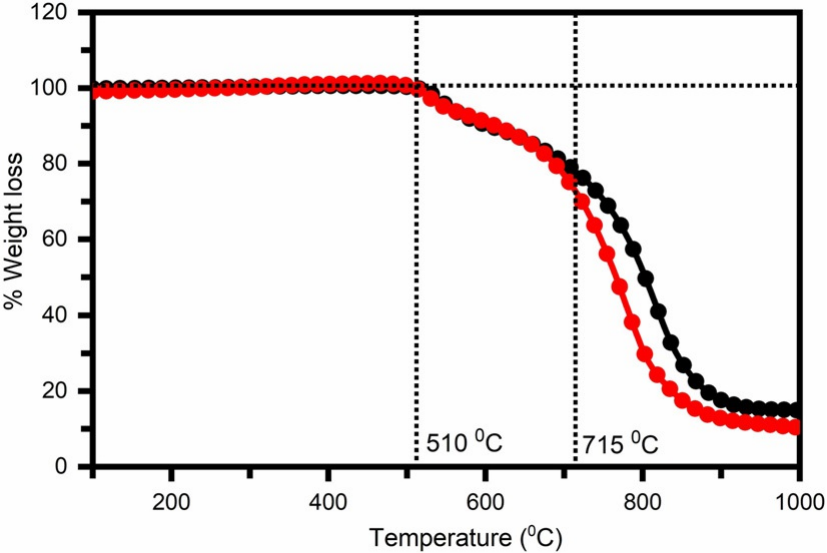
Thermo‐gravimetric analysis (TGA) of undoped and Fe‐doped Cs_2_AgBiCl_6_ double perovskites for testing the thermal stability.

The thermal and chemical stability of perovskite materials is an important parameter to evaluate their ability for device applications. To investigate the thermal stability of Fe‐doped Cs_2_AgBiCl_6_ perovskite, TGA was carried out. Temperature‐dependent weight loss of the undoped and Fe‐doped Cs_2_AgBiCl_6_ double perovskites is shown in Figure 2. As seen, the weight loss of the Fe‐doped double perovskites is found in two steps. The first step of weight loss starts around 510–715 °C with 22 wt % and the second significant weight loss is observed at about 78 wt %, which mainly takes place from 715–1000 °C. It can be seen from the thermo‐gram that the Fe‐doped Cs_2_AgBiCl_6_ perovskite are highly stable until 510 °C and the decomposition starts above 510 °C. This result indicates that Fe‐doped Cs_2_AgBiCl_6_ double perovskites have excellent thermal stability.

### X‐ray photoelectron spectroscopy (XPS) analysis

**Figure 3:**
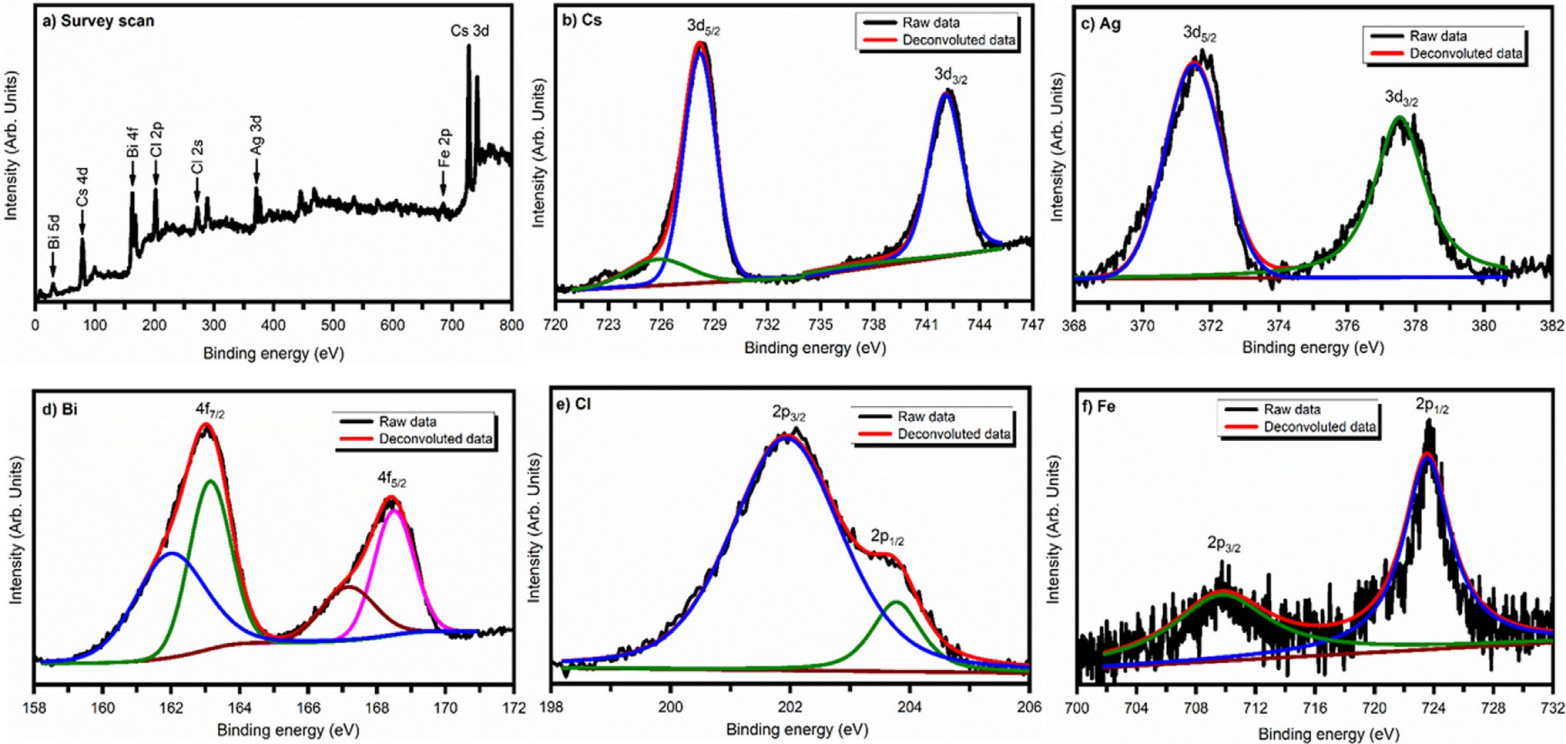
XPS spectra of Fe‐doped Cs_2_AgBiCl_6_double perovskite (a) Survey scan from 0–800 eV, (b) Narrow scan for Cs 3d in the range 720–747 eV, (c) Narrow scan for Ag 3d in the range 368–382 eV, (d) Narrow scan for Bi 4f in the range 158–172 eV, (e) Narrow scan for Cl2p in the range 198–206 eV and (f) Narrow scan for Fe2p in the range 700–732 eV.

Quantitative analysis of the electronic structures and chemical properties of Fe‐doped Cs_2_AgBiCl_6_ double perovskite has been performed by XPS analysis. Figure 3 a illustrates the survey and high‐resolution XPS spectra of Fe‐doped Cs_2_AgBiCl_6_ double perovskite. As seen, the peaks corresponding to cesium [Cs(3d)], silver [Ag (3d)], bismuth [Bi (4f)], chlorine [Cl (2p)] and iron [Fe (2p)] appear in the Fe‐doped Cs_2_AgBiCl_6_ double perovskite. Figure 3 b–f are the narrow scan XPS spectra for 3d‐Cs, 3d‐Ag, 4f‐Bi, 2p‐Cl and 2p‐Fe elements, respectively. In Figure 3 b, two strong peaks were observed for the narrow XPS spectra of 3d‐Cs. The peak at 728.1 eV is due to 3d_5/2_‐Cs and peak at 742.1 eV due to 3d_3/2_‐Cs consistent with the standard Cs element. The two peaks were disjoint with an energy value of 14.0 eV. The 3d‐Ag spectra consist of two peaks at 371.7 and 377.6 eV associated with Ag 3d_5/2_ and Ag 3d_3/2_, respectively. The core‐level peaks for Bi are found at the binding energy 163.0 eV due to Bi 4f_7/2_ and 168.5 eV due to 4f_5/2_. The energy separation between these two peaks was measured 5.5 eV, which is a characteristic signal from the Bi^3+^ species (Figure 3 d). The 2p‐Cl spectra composed of two peaks at 202.0 and 203.7 eV originating from Cl 2p_3/2_ and Cl 2p_1/2_, respectively (Figure 3 e). Figure 3 f shows the 2p‐Fe XPS spectra of the Fe‐doped Cs_2_AgBiCl_6_ double perovskite material. The 2p‐Fe has two peaks with binding energies 710.78 and 724.10 eV which are related to Fe 2p_3/2_ and 2p_1/2_, respectively. The binding energy of all the elements is slightly shifted at higher values than the reported one, demonstrating that the stronger M‐Cl interaction in the [BiCl_6_]^3−^ and [AgCl_6_]^5−^ octahedra and leads to differing the chemical environment around Bi, Ag, and Cs on Fe‐incorporation. The results are analogous to that of earlier reported systems.[^46^, ^47^, ^48^] These results confirm the effective doping of Fe in Cs_2_AgBiCl_6_ double perovskite material. The XPS spectra of the pristine Cs_2_AgBiCl_6_ and 3 % Fe‐doped double perovskite are provided in Figures S1 and S2 in the Supporting Information.

### Electron microscopy analysis

**Figure 4:**
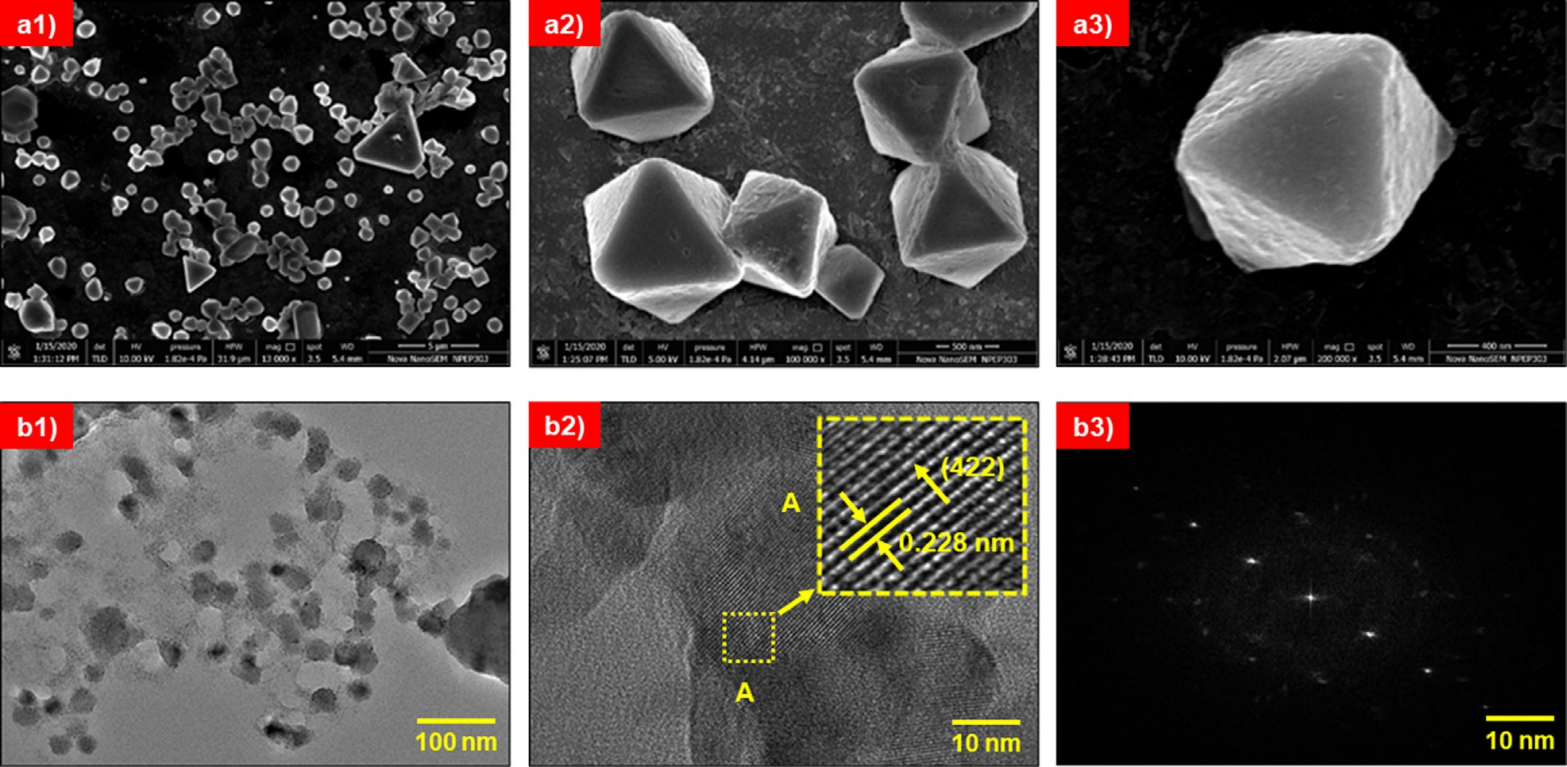
(a1), (a2) and (a3) Field emission scanning electron microscopy (FE‐SEM) images of Fe‐doped Cs_2_AgBiCl_6_ perovskite at different resolutions. Transmission electron microscope (LE‐TEM) image of Fe‐doped Cs_2_AgBiCl_6_ perovskite (b1) Low resolution (b2) High resolution. The inset is the enlarged view of area ′A′ of HR‐TEM image, and (b3) Selected area electron diffraction (SAED) pattern of Fe‐doped Cs_2_AgBiCl_6_ perovskite.

Field emission scanning electron microscope (FE‐SEM) and transmission electron microscope (TEM) were used to study surface morphology and topography of the Fe‐doped Cs_2_AgBiCl_6_ double perovskite. The FE‐SEM images of the Fe‐doped Cs_2_AgBiCl_6_ double perovskite at different resolutions are shown in Figure 4 a1–a3. It shows the formation of micrometer‐sized multifaceted crystals, including several uniform dipyramidal units having a size of 1–2 μm. A close‐up picture in the magnified images for one microcrystal (Figure 4 c) shows perfect dipyramidal units with a cubic crystal system. The fine faceted microcrystal confirmed the double perovskite material is formed with high crystallinity.

Figure 4 b1 and Figure 4 b2 shows low‐resolution transmission electron microscopy (LR‐TEM) and high‐resolution TEM (HR‐TEM) images of the Fe‐doped Cs_2_AgBiCl_6_ double perovskites, respectively. The inset in Figure 4 b2 is an enlarged area “A” view of the HR‐TEM image, which shows that the inter‐planar spacing between two adjacent lattice planes of the Fe‐doped Cs_2_AgBiCl_6_ perovskite to be 0.228 nm, which is consistent with the spacing of the (422) plane of Fe‐doped Cs_2_AgBiCl_6_ double perovskite crystal. These results are also consistent with the X‐ray diffraction results (see Table S3). Figure 4 b3 is the selected area electron diffraction (SAED) pattern of Fe‐doped Cs_2_AgBiCl_6_ double perovskite. The bright diffraction spots in the SAED reveal the polycrystalline nature of the Fe‐doped Cs_2_AgBiCl_6_ perovskite with a high degree of crystallinity.

### Energy‐dispersive X‐ray spectroscopy (EDS) analysis

**Figure 5:**
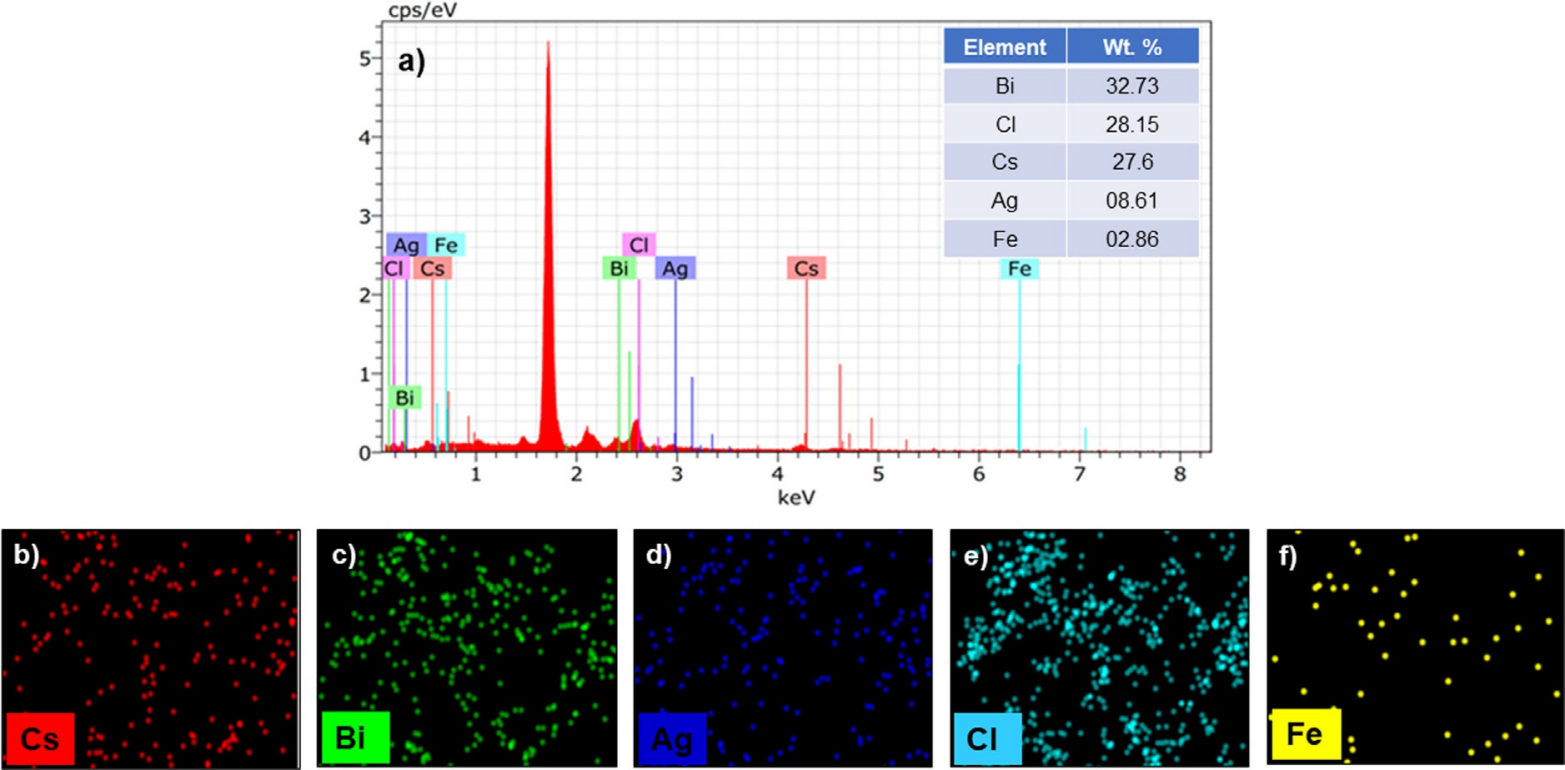
A typical EDS spectrum recorded for Fe‐doped Cs_2_AgBiCl_6_ perovskite. The mapping shows the overlapping of Cs, Bi, Ag, Fe, and Cl elements and the homogenous distribution of Fe in the entire Cs_2_AgBiCl_6_ perovskite lattice.

The compositional analysis of Fe‐doped Cs_2_AgBiCl_6_ double perovskite was carried out using Energy Dispersive X‐ray Analysis (EDS) technique. A typical EDS spectrum recorded in the binding energy region of 0–10 KeV for the Fe‐doped Cs_2_AgBiCl_6_ double perovskite is shown in Figure 5 a. The EDS mapping (Figure 5 b, 5 c, 5 d, 5 e, and 5 f) shows the overlapping of Cs, Ag, Bi, Cl, and Fe elements, respectively. It indicates that Fe is homogeneously incorporated in the entire Cs_2_AgBiCl_6_ double perovskite lattice. EDS pattern of the elements displays peaks at 0.7 and at 6.4 keV, which confirms the presence of Fe in the crystal lattice.

### Optical properties

**Figure 6:**
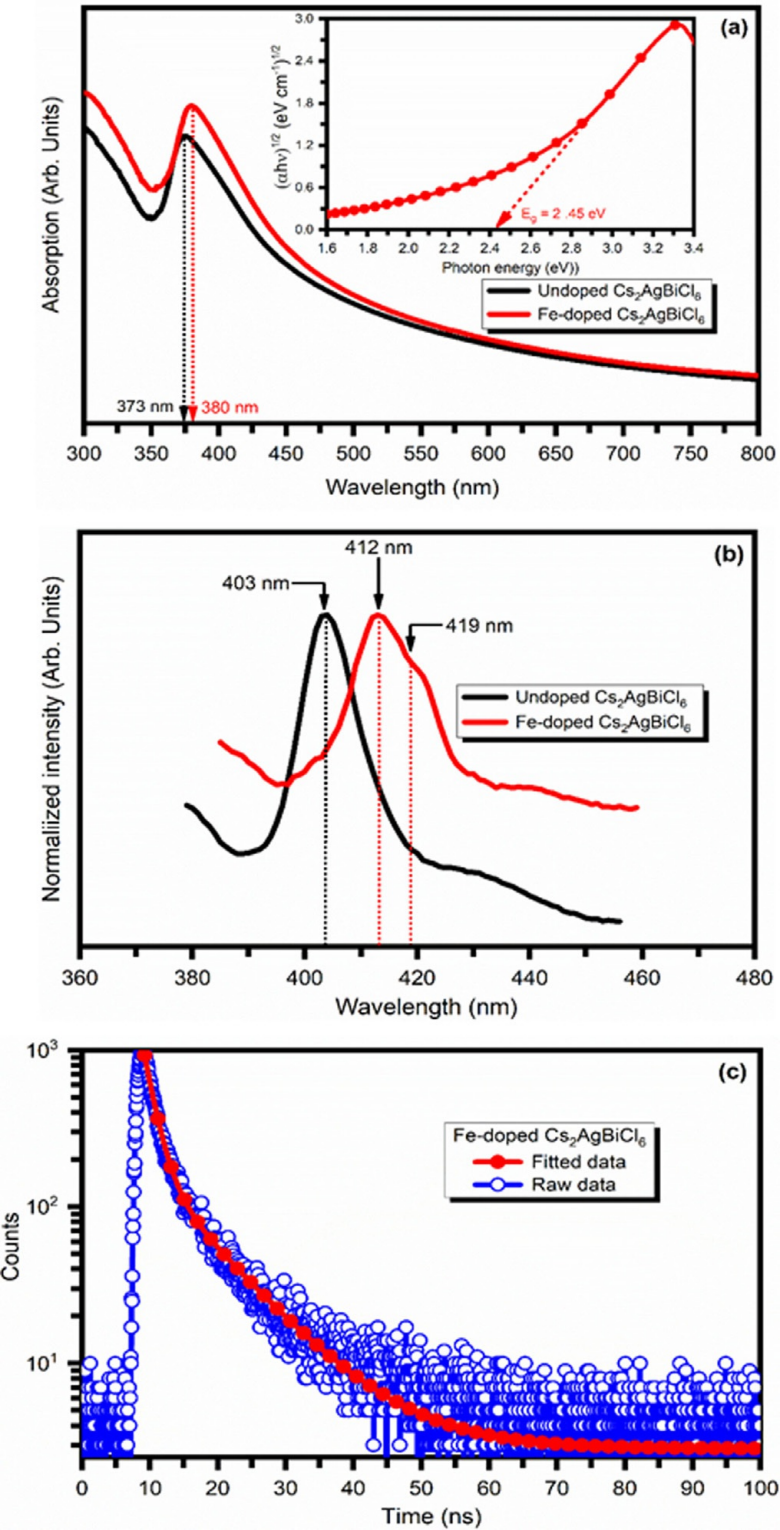
(a) UV/Visible absorption spectra of undoped and Fe‐doped Cs_2_AgBiCl_6_ double perovskites. Inset is the typical Tauc's plot used to estimate optical band gap (b) Photoluminescence (PL) spectra of Fe‐doped Cs_2_AgBiCl_6_ double perovskite (c) Time‐resolved PL decays.

UV/Visible absorption spectroscopy was used to investigate the optical properties and light absorption capability of the Fe‐doped Cs_2_AgBiCl_6_ double perovskite. Figure 6 a shows the room‐temperature absorption spectra of the undoped and Fe‐doped Cs_2_AgBiCl_6_ double perovskite. Fe‐doped Cs_2_AgBiCl_6_ double perovskite samples reveal a sharp absorption peak with the first electronic transition peak at 373 nm (3.31 eV) and 380 nm (3.27 eV). The absorption peak also shows a long tail extended to 700 nm, which suggests that the transition from sub‐band gap states may originate from the surface defect of the Fe‐doped Cs_2_AgBiCl_6_ double perovskite material. The absorption spectra of the Fe‐doped Cs_2_AgBiCl_6_ double perovskite reveal a small change in peak positions and shapes. It is consistent with the point that the 6s→6p transition of the Bi^3+^ ions largely directed the first electronic transition of Fe‐doped Cs_2_AgBiCl_6_ microcrystal.[^20^, ^37^] The absorption peak suggests negligible perturbations in the electronic structure of Cs_2_AgBiCl_6_ when Fe^3+^ is incorporated into the lattice. The UV/Visible spectra are used to estimate the optical band gap of Fe‐doped Cs_2_AgBiCl_6_ perovskite. Absorbance and incident photon energy can be directly related using equation,^[49]^

(1) $${\left(\alpha hv\right)}^{1/n}=C\;\left(hv-E_{g}\right)\;$$

in which *h* is Planck's constant, α is the absorption coefficient, *ν* is the frequency of light, C is the proportionality constant, *E*_g_ is the band gap, and *n* is integer 1/2 or 2 depending on the material and whether it is a direct or indirect band gap. The inset of Figure 6 a shows the typical Tauc's plot for Fe‐doped Cs_2_AgBiCl_6_ double perovskite used for the estimation of the optical band gap. The indirect band gap of the Fe‐doped Cs_2_AgBiCl_6_ double perovskite is estimated at 2.45 eV, whereas the pristine Cs_2_AgBiCl_6_ has an indirect band gap of 2.77 eV. The band gap reduction after Fe‐doping may be due to the overlap of the Ag‐d/Cl‐p orbital and the Fe‐3d orbital during the construction of the valence band and new VBM shifting towards a higher energy level as revealed by DFT calculations (Figure 8). A similar result has been observed in Cu‐doped Cs_2_AgInCl_6_, Sb‐doped Cs_2_AgBiBr_6_ and Ti‐doped Cs_2_AgBiBr_6_.[^48^, ^50^] The decrease in band gap suggests that the Fe‐doped Cs_2_AgBiBr_6_ double perovskite material is a more suitable absorber for tandem solar device applications. We examined the photophysical properties and determined the Urbach energy (*E*_u_) of Fe‐doped double perovskite materials. It shows structural disorders, effects of impurities, and electron‐phonon interactions in the absorption phenomenon.^[51]^

The Urbach energy is estimated by using,^[52]^

(2) $$\alpha =\alpha_{o\;}exp\left(\frac{hv}{E_{u}}\right)$$

in which α is the absorption coefficient, and *hv* is the photon energy. The Urbach energy is then estimated by plotting ln(α) as a function of (*hv*) shown in Figure S3 (Supporting Information). The reciprocal of the slope of a linear fit gives the value of Urbach energy. The Urbach energy for the undoped and Fe‐doped Cs_2_AgBiCl_6_ is found to be 1.2 eV and 1.0 eV, respectively.

The room temperature photoluminescence (PL) spectrum of the undoped and Fe‐doped Cs_2_AgBiCl_6_ double perovskites excited at a wavelength of 360 nm is shown in Figure 6 b. As can be seen, the Fe‐doped Cs_2_AgBiCl_6_ double perovskite exhibit a major PL peak at 412 nm and a tiny shoulder (419 nm), and these appear as a result of band edge emission and sub‐band gap trap states emission, respectively. The major PL peak is red shifted by 9 nm from the undoped Cs_2_AgBiCl_6_ perovskite. It is interesting to note that the band gap values obtained using the PL spectrum are slightly higher than those obtained from the UV/Visible spectrum. It can be attributed to the band‐edge excitonic irradiative luminescence.

To get further insights into the optical properties of doped Cs_2_AgBiCl_6_ double perovskite material, time‐resolved photoluminescence (TRPL) analysis has been carried out. Figure 6 c shows the time‐resolved photoluminescence emission spectra for Fe‐doped Cs_2_AgBiCl_6_ double perovskite materials in isopropanol using a laser excitation wavelength of 412 nm. The TRPL traces at 412 nm wavelength show an initially fast decay, followed by a slow decay with a tail. The PL decay time of the Fe‐doped Cs_2_AgBiCl_6_ double perovskite is reduced as compared to the undoped Cs_2_AgBiCl_6_ double perovskite materials.^[37]^ It may be the enhancement in the non‐radiative recombination rate.^[48]^ The emission decay traces can be well fitted to bi‐exponential function,[^53^, ^54^, ^55^]

(3) $$A\left(t\right)=A_{1}\exp\left(-\frac{t}{\tau_{1}}\right)+A_{2}exp\left(-\frac{t}{\tau_{2}}\right)$$

in which τ_1_ and τ_2_ are the decay constants for the fast and slow components of the traces, respectively. The short‐lived component (t_1_) and a long‐lived component (t_2_) are estimated at 1.71 ns and 9.41 ns at 412 nm excitation wavelength. The calculated kinetic parameters are displayed in Table 2. The average carrier lifetime for the Fe‐doped Cs_2_AgBiCl_6_ double perovskite at excitation wavelength 412 nm is in the order of 8.41 ns. It is reported that the biexponential decay corresponds to two different phenomena. First, the short life‐time PL component is due to the recombination of initially generated excitons, while the second is the long‐life time PL attributed to recombination of exciton during the contribution of surface states, which act as stable excitons at room temperature.^[56]^ However, more investigation is necessary to get additional insights into the phenomenon of exciton dynamic.

**Table 2:** Photo‐physical data of Fe‐doped Cs_2_AgBiCl_6_ double perovskite material.

Fe‐doped Cs_2_AgBiCl_6_	τ_1_ [ns]	α_1_ [%]	τ_2_ [ns]	α_2_ [%]	CHI SQ	τ_ave_ [ns]
At 412 nm	1.71	44.98	9.41	55.02	1.18	8.41

### Photo‐response properties

**Figure 7:**
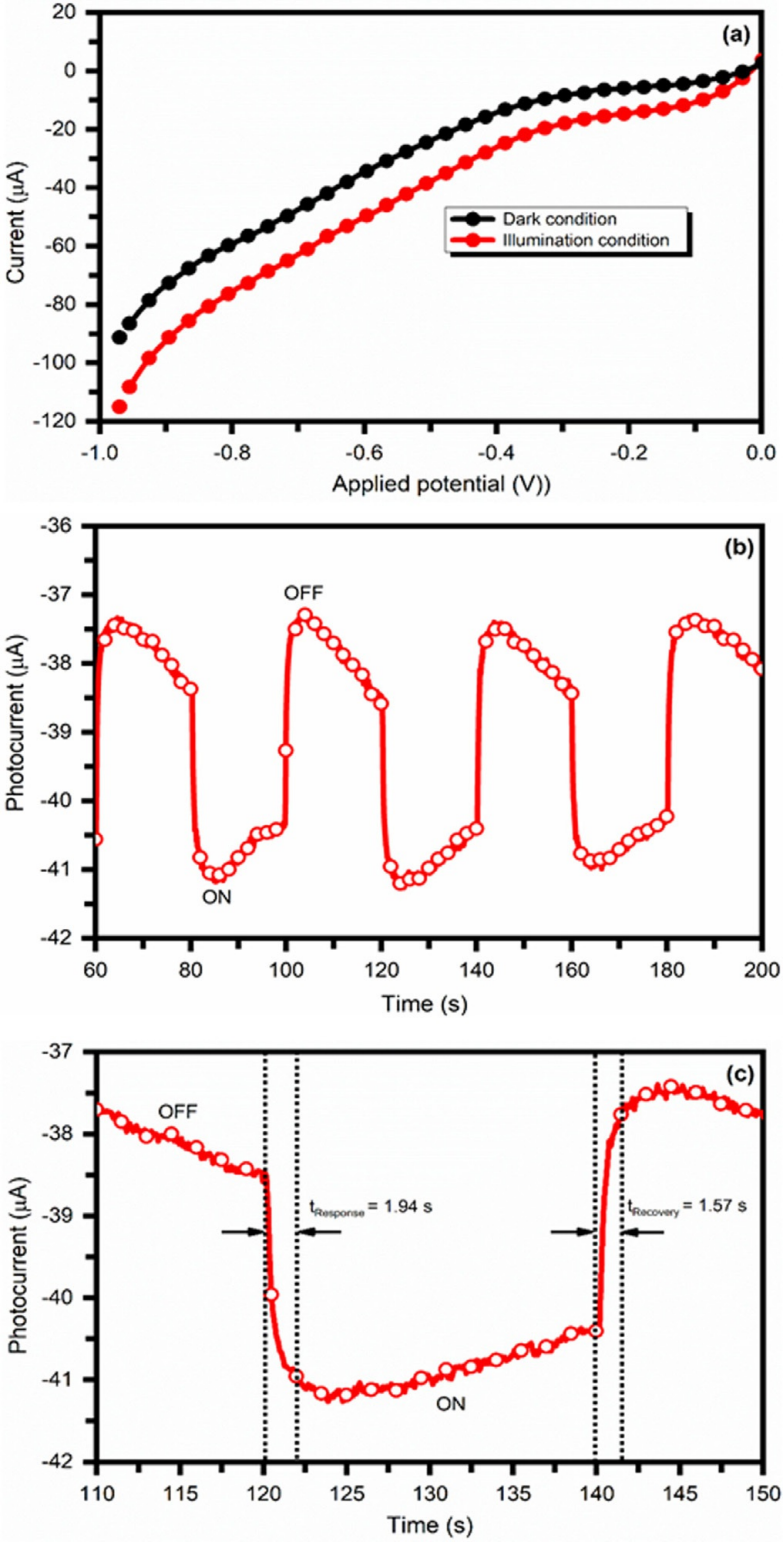
(a) Current density‐voltage (*J–V*) curves for Fe‐doped Cs_2_AgBiCl_6_ double perovskite under dark and visible light illumination, (b) Time‐dependent current response of Fe‐doped Cs_2_AgBiCl_6_ double perovskite for repeated cycles under dark and white light illumination at a constant bias voltage of −0.4 V and (c) Single‐cycle of time‐dependent current response of Fe‐doped Cs_2_AgBiCl_6_ double perovskite *J–T* curve using white light illumination to measure the response and recovery time of charge carriers.

The photoelectrochemical (PCE) experimentation of Fe‐doped Cs_2_AgBiCl_6_ double perovskite was carried out using linear sweep current density–voltage (*J–V*) technique under dark and light visible light irradiation. Figure 7 a shows the current density–voltage (*J–V*) curves for the Fe‐doped Cs_2_AgBiCl_6_ double perovskite under dark and visible light illumination. A significant increase in the negative current under visible light illumination for the Fe‐doped Cs_2_AgBiCl_6_ double perovskite is observed. Figure 7 b shows the time‐dependent current response of the Fe‐doped Cs_2_AgBiCl_6_ double perovskite for repeated cycles under dark and white light illumination at a constant bias voltage −0.4 V. Sharp rise and decay of the photocurrent has been observed when the light is turned ON and OFF. The rapid increase of photocurrent density 3 μA cm^−2^ has been observed after illumination for Fe‐doped Cs_2_AgBiCl_6_ double perovskite photoelectrode and is recovered rapidly in dark conditions. Figure 7 c shows a single‐cycle of time‐dependent current response of the Fe‐doped Cs_2_AgBiCl_6_ double perovskite *J–T* curve using white light illumination to measure the response and recovery time of charge carriers. The response and recovery time for the charge carrier of Fe‐doped Cs_2_AgBiCl_6_ double perovskite material is found 1.94 s and 1.57 s, respectively. The fast response and recovery of charge carrier for the Fe‐doped Cs_2_AgBiCl_6_ double perovskite thin films under white light illumination make them suitable for various opto‐electronics applications, photocatalysts, and photovoltaics as environmentally friendly halide double perovskites.

### Density functional theory (DFT) analysis

**Figure 8:**
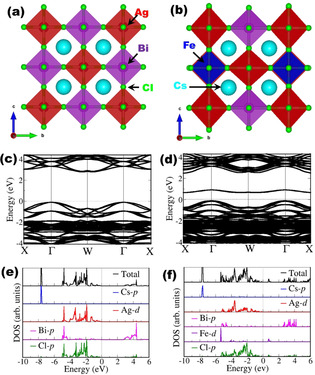
Cubic crystal structure of (a) Cs_2_AgBiCI_6_ perovskite, (b) Fe‐doped Cs_2_AgBiCI_6_ perovskite in polyhedral presentation (c) and (d) Band structures of Cs_2_AgBiCI_6_ and Fe‐doped Cs_2_AgBiCI_6_ perovskites along high‐symmetry directions of the Brillouin zone, (e) and (f) Partial density of states (PDOS) of Cs_2_AgBiCI_6_ and Fe‐doped Cs_2_AgBiCI_6_ perovskites predicted with HSE06 functional.

The experimental results were corroborated by first‐principles density functional theory (DFT) calculations. The optimized structures of the undoped and Fe‐doped Cs_2_AgBiCI_6_ materials in the cubic crystal structure (*Fm* $\bar{3}$ *m* space group) are shown in Figure 8. The lattice parameters are 10.885 Å for pristine Cs_2_AgBiCI_6_ and 10.782 Å for the Fe‐doped Cs_2_AgBiCI_6_, which indicate that Fe substitutional doping at Bi site results in contraction of the lattice, owing to the smaller ionic radius of Fe^3+^ ion (0.63 Å) than Bi^3+^ ion (1.03 Å). The calculated lattice parameters are consistent with the values estimated by XRD analysis. The band structures (Figure 8 c and Figure 8 d) reveal indirect band gaps, predicted at 2.75 eV and 2.51 eV, respectively, for the undoped and Fe‐doped Cs_2_AgBiCI_6_ double perovskites. The DFT predicted band gap values for undoped and Fe‐doped Cs_2_AgBiCl_6_ double perovskites are consistent with those obtained from UV/Visible spectroscopy analysis. The substitution of Bi by Fe resulted in the introduction of donor states close to the top of the valence band. The partial density of states (PDOS) plots (Figure 8 e and Figure 8 f) shows that the valence band to be composed mainly of CI‐p orbitals, whereas the conduction band is composed more of Bi‐p orbitals.

## Conclusions

We have successfully synthesized Fe‐doped Cs_2_AgBiCl_6_ double perovskite that displays blue emission via an antisolvent method. The formation of Fe‐doped Cs_2_AgBiCl_6_ double perovskite is confirmed by XRD and XPS analyses. XRD pattern of the Cs_2_AgBiCl_6_ perovskite recorded after six months of exposure to ambient environmental conditions shows that the material has high structural stability. Also, TGA results indicate that both Fe‐doped Cs_2_AgBiCl_6_ perovskites have high thermal stability (510 °C). FE‐SEM analysis revealed the formation of dipyramidal in shape Fe‐doped Cs_2_AgBiCl_6_ double perovskite and EDS mapping shows the overlapping of Cs, Bi, Ag, Fe, and Cl elements, with the Fe homogeneously incorporated in the entire Cs_2_AgBiCl_6_ perovskite lattice. The Fe‐doped Cs_2_AgBiCl_6_ double perovskite shows a sharp absorption peak at 380 nm and extends up to 700 nm suggesting the transition from sub‐band gap states may originate from the surface defect of Fe‐doped Cs_2_AgBiCl_6_ perovskite material. Lattice parameters and band gap values of the Fe‐doped Cs_2_AgBiCl_6_ double perovskites predicted by the DFT calculations are confirmed by XRD and UV/Visible spectroscopy analysis. Finally, time‐dependent photoresponse characteristics for Fe‐doped Cs_2_AgBiCl_6_ double perovskite show fast response and recovery time of charge carriers. The displayed high thermal stability and excellent response and recovery time of charge carriers in the Fe‐doped Cs_2_AgBiCl_6_ double perovskite make it a suitable lead‐free solar absorber for various opto‐electronics applications, including photocatalysis and photovoltaics.

## Conflict of interest

The authors declare no conflict of interest.

## Supporting information

As a service to our authors and readers, this journal provides supporting information supplied by the authors. Such materials are peer reviewed and may be re‐organized for online delivery, but are not copy‐edited or typeset. Technical support issues arising from supporting information (other than missing files) should be addressed to the authors.

Supplementary
