# Supplementary material for: Experimental and Theoretical Investigation of the Structural and Opto‐electronic Properties of Fe‐Doped Lead‐Free Cs2AgBiCl6 Double Perovskite
Source: Chemistry. 2021 Mar 22;27(26):7408–17. doi: 10.1002/chem.202004902 (PMC8252727; doi:10.1002/chem.202004902)

Chemistry–A European Journal

Supporting Information

Experimental and Theoretical Investigation of the Structural and Opto-electronic Properties of Fe-Doped Lead-Free $Cs_2AgBiCl_6$ Double Perovskite

Sachin Thawarkar,*[a] Sachin R. Rondiya,[b] Nelson Y. Dzade,[b] Nageshwar Khupse,[c] and Sandesh Jadkar*[a]

SCHEME S1: Schematic of PEC cell employed in the present study for the study of photo response properties of Fe-doped $Cs_2AgBiCl_6$ double perovskite

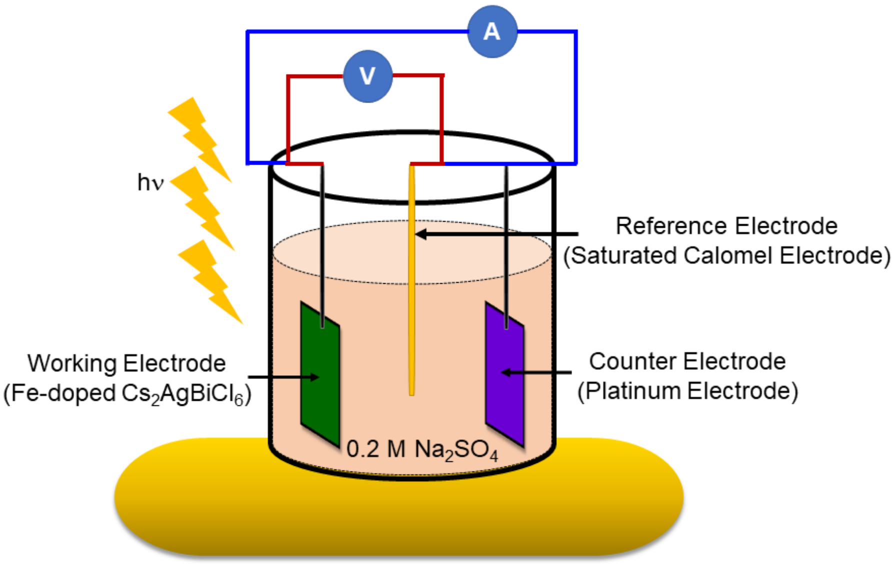

SCHEME S2: Schematic representation and the synthesis protocol used for the synthesis of undoped and Fe doped $Cs_2AgBiCl_6$ double perovskites by anti-solvent method. Optical images of a colloidal solution of Fe-doped $Cs_2AgBiCl_6$ double perovskite **a)** Under daylight and **b)** 365 nm UV light excitation.

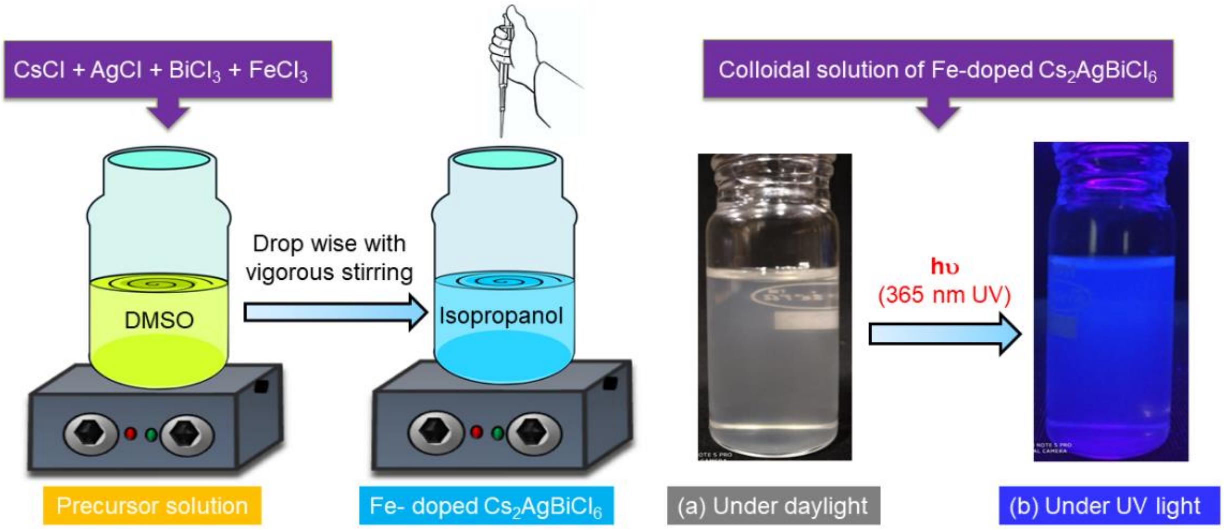

Table S1: The interplanar d-spacings values (d) and corresponding diffraction peak positions (2θ) for different diffraction planes (hkl) for Fe-doped $Cs_2AgBiCl_6$ double perovskite

(hkl) plane	Undoped Fe-doped $Cs_2AgBiCl_6$		3 % Fe-doped $Cs_2AgBiCl_6$		6 % Fe-doped $Cs_2AgBiCl_6$	
	**2θ**	**d (nm)**	**2θ**	**d (nm)**	**2θ**	**d (nm)**
**(220)**	22.83°	0.389	23.63°	0.376	23.84°	0.373
**(400)**	32.76°	0.273	33.36°	0.268	33.72°	0.265
**(422)**	40.51°	0.222	41.27°	0.219	41.47°	0.217
**(440)**	47.24°	0.192	47.98°	0.189	48.19°	0.188
**(620)**	53.29°	0.171	54.00°	0.170	54.25°	0.168
**(444)**	58.91°	0.156	59.60°	0.155	59.89°	0.154
**---**	64.22°	0.144	64.92°	0.143	65.12°	0.143
**---**	69.41°	0.135	70.02°	0.134	70.22°	0.133
**---**	74.27°	0.127	74.93°	0.127	75.23°	0.123

FIGURE S1: XPS spectra of prestine $Cs_2AgBiCl_6$ double perovskite **(a)** Survey scan from 0-800 eV,**(b)** Narrow scan for Cs 3d **(c)** Narrow scan for Ag 3d **(d)** Narrow scan for Bi 4f **(e)** Narrow scan for Cl2p

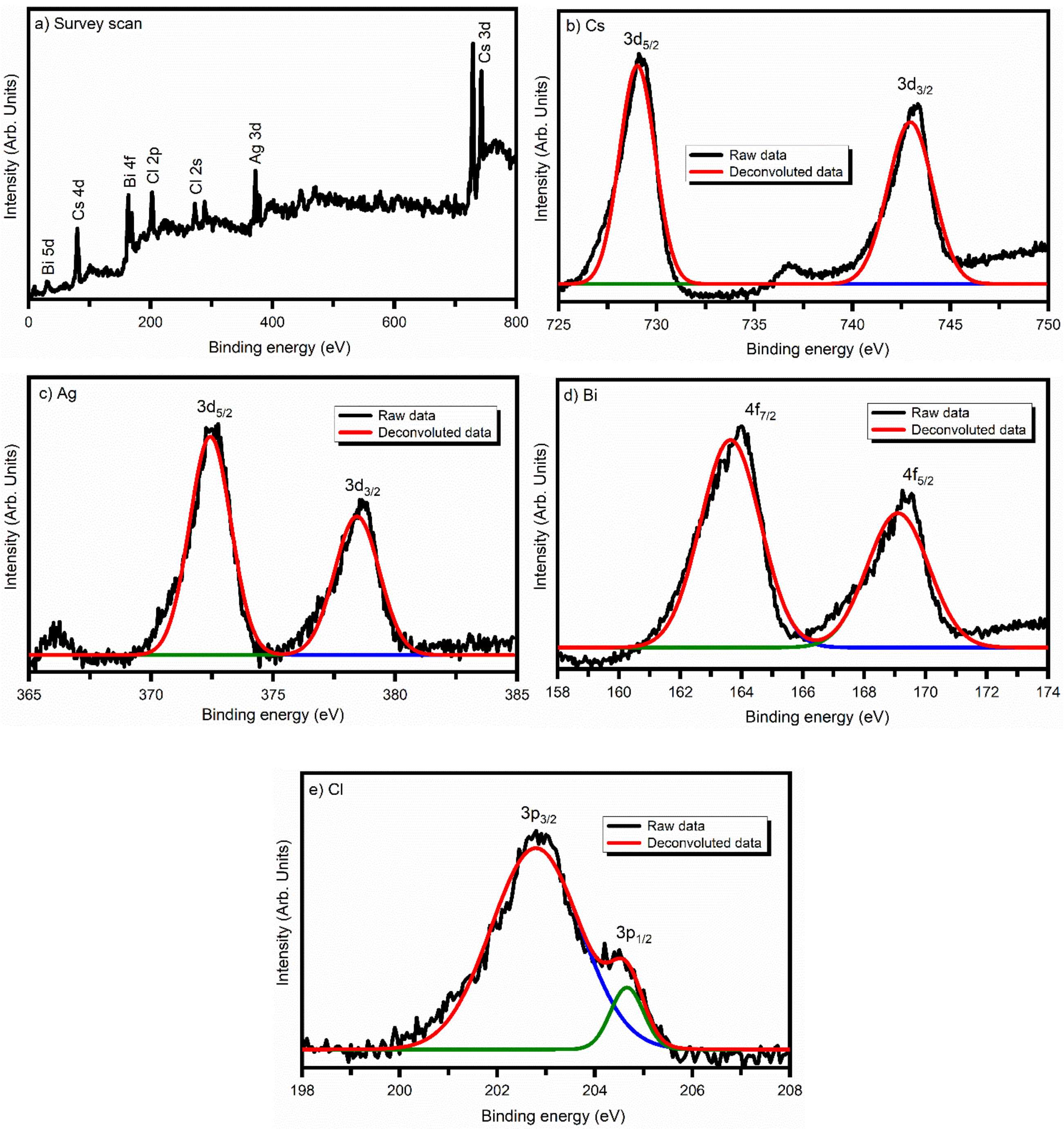

FIGURE S2: XPS spectra of 3 % Fe-doped $Cs_2AgBiCl_6$ double perovskite **(a)** Survey scan from 0-800 eV, **(b)** Narrow scan for Cs 3d, **(c)** Narrow scan for Ag 3d, **(d)** Narrow scan for Bi 4f **(e)** Narrow scan for Cl2p and **(f)** Narrow scan for Fe2p

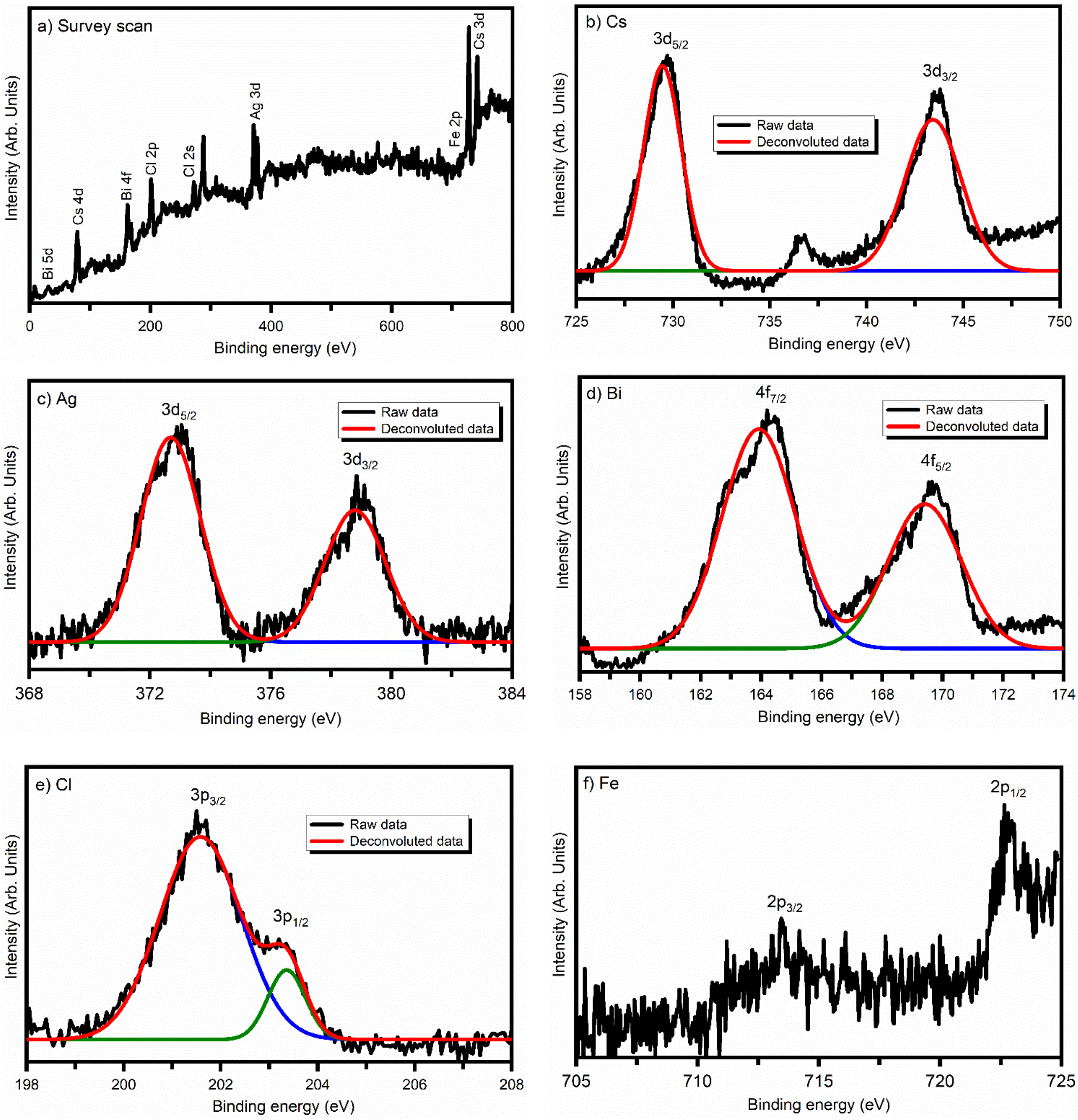

FIGURE S3: Plot of Urbach energy for **a)** Undoped $Cs_2AgBiCl_6$ and **b)** Fe-doped $Cs_2AgBiCl_6$ perovskite

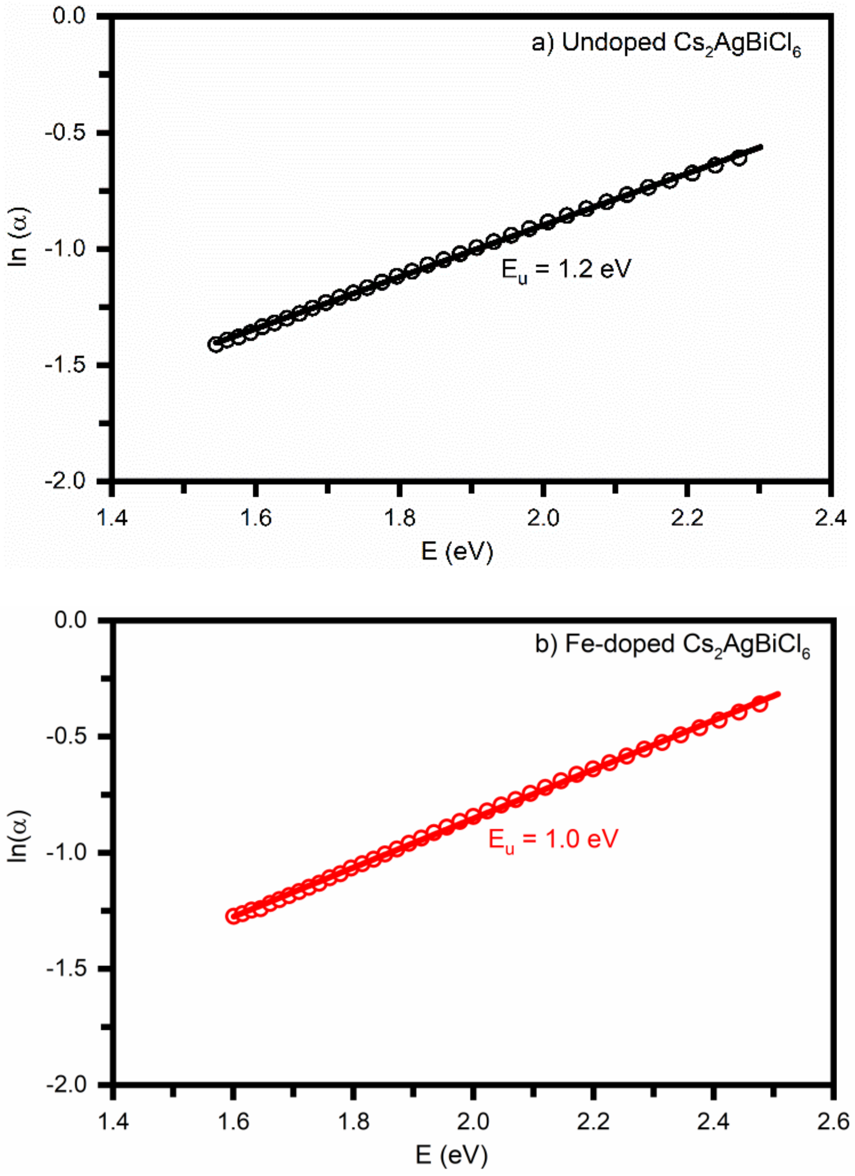

Supplement: Supplementary file 1 — Supplementary [file CHEM-27-7408-s001.pdf]
